# Meta-Analysis of Randomized Clinical Trials Evaluating Effectiveness of a Multivitamin Supplementation against Oxidative Stress in Healthy Subjects

**DOI:** 10.3390/nu14061170

**Published:** 2022-03-10

**Authors:** Seoyoung Lee, Iksoo Huh, Seunghee Kang, Yea-eun Nam, Youngseo Cho, Md Kamruzzaman, Jina Hong, Oran Kwon, Taesung Park

**Affiliations:** 1 College of Liberal Studies, Department of Liberal Studies, Seoul National University, Seoul 08826, Korea; seoyoung1215@snu.ac.kr; 2 College of Nursing, Research Institute of Nursing Science, Seoul National University, Seoul 03080, Korea; huhixoo@gmail.com; 3 Graduate Program in System Health Science and Engineering, Department of Nutritional Science and Food Management, Ewha Womans University, Seoul 03760, Korea; nutrishee@gmail.com (S.K.); yeaeun@ewhain.net (Y.-e.N.); 4 Department of Statistics, Seoul National University, Seoul 08826, Korea; 14choyoung@naver.com (Y.C.); kzaman1@isrt.ac.bd (M.K.); 5 Access Business Group International, LLC, 5600 Beach Blvd., Buena Park, CA 90621, USA; jina.hong@amway.com

**Keywords:** meta-analysis, multivitamin supplementation, exogenous oxidant scavenger, oxidative damage

## Abstract

A meta-analysis has been widely applied to draw general conclusions using a set of studies with similar purposes and designs. This study aimed to perform a meta-analysis of six randomized placebo-controlled trials, independently conducted for the relationship between a plant-based multivitamin/mineral supplementation (PMS) and oxidative stress for 6 to 8 weeks, to provide overall estimates of those effects. In detail, linear mixed model analysis was first conducted on each study to obtain individual estimates; then, two types of meta-analysis were applied to combine the individual estimates from all available studies (overall meta-analysis) and region-specific studies (subgroup meta-analysis). In the meta-analysis, we selected 19 biomarker variables that overlapped in at least two studies and found 6 variables significant in at least one meta-analysis. The overall estimates of beta coefficients were 0.17 for vitamin C, 0.80 for vitamin B_6_, 0.46 for vitamin B_12_, 0.81 for folate, 0.36 for *β*-carotene, and −0.17 for oxidized LDL (ox-LDL). Subsequent association analysis revealed significant negative correlations between plasma free radical scavenging nutrients and plasma ox-LDL levels, indicating a general benefit of PMS in alleviating oxidative stress by providing exogenous oxidant scavengers.

## 1. Introduction

Oxidative stress is an imbalance between the generation of reactive oxygen species (ROS) and the ability to deactivate them. The association of oxidative stress with many chronic diseases has been extensively studied and found to be an essential factor in inducing public health problems in developed and developing countries [1]. Therefore, an array of clinical trials has been performed to prove that taking an antioxidant multivitamin supplement may affect reducing the risks of chronic diseases [2,3,4,5,6]. However, the clinical trials generally have involved several complex features and have suffered from a small sample size, thus reporting inconsistent findings on functional and physiological levels [7,8,9].

Such consistency and inconsistency have also been found in randomized and placebo-controlled trials (RCTs), which were independently conducted to evaluate the effects of the same plant-based multivitamin supplement (PMS) on reducing oxidative damage, maintaining endogenous ROS homeostasis, or providing heart health benefits in general. The PMS contained nutritional doses of multiple vitamins/minerals and phytonutrients from extracts or powders of various fruits and vegetables. Similar protocols were used but on regionally different populations. The directions of the results were generally consistent; however, not every individual study detected statistical significance, probably due to the relatively small sample size [10]. In addition, individual studies adopted various variables under different experimental conditions. Moreover, sometimes, slight bias, such as recall bias in an intervention study, may reduce accuracy, leading to contradictive results [11]. Consequently, inconsistent statistical results obtained from individual studies made it difficult to conclude the general impact of PMS supplementation on oxidative stress.

Meta-analysis is a set of statistical procedures synthesizing data by combining results from independent studies to integrate the findings and provide overall estimates of the relationship between variables [12]. It has advantages over conventional narrative review in that it can focus on the magnitude of the general effect of interest rather than the statistical significance of individual studies. In addition, it uses a greater number of samples than in single studies, providing a smaller standard error and *p*-value. Thus, it is expected to provide better estimation results than single studies. However, conducting a meta-analysis is impossible if the extracted studies do not contain enough information of parameter estimates, such as beta coefficients or standard error of interest [13].

Therefore, the current study aimed to perform a meta-analysis based on the previously performed RCTs of PMS to shed light on the controversy and reach a general conclusion about its effectiveness in maintaining health by reducing oxidative stress. In detail, we collected individual subject datasets of six (three published [14,15,16] and three unpublished) RCTs incorporating 886 participants and aggregated the effect sizes. As variables of interest, we selected 19 biological marker variables that are overlapped in at least two studies and applied meta-analysis, considering the heterogeneity of effects from PMS supplementation. Furthermore, in addition to the overall meta-analysis that uses all available studies, a subgroup meta-analysis was undertaken to analyze the differences in regional affiliation of the participants.

## 2. Materials and Methods

### 2.1. Test Samples

The PMS sample provided nutritional doses of 14 vitamins (vitamin A, B_1_, B_2_, B_6_, B_12_, C, D, E, K, *β*-carotene, biotin, folate, niacin, and pantothenic acid) and 10 minerals (Ca, Cr, Cu, I, Fe, Mn, Mg, Mo, Se, and Zn). In addition, phytonutrients were provided from the fruits and vegetables, including acerola, alfalfa, apple, bilberry, black currant, blueberry, brassica, broccoli, carrot, citrus, cranberry, elderberry, grape, grapefruit, horseradish, kelp, lemon, mandarin orange, marigold, onion, orange, parsley, peppermint, plum, rosemary, spinach, tomato, turmeric, and watercress. The placebo samples were formulated to match the shape and color of the PMS sample, primarily using dextrose, microcrystalline cellulose, silicon dioxide, croscarmellose sodium, and magnesium.

### 2.2. Study Collection and Variable Selection

Six independent RCTs investigating the effectiveness of PMS against oxidative stress were collected for this meta-analysis (Table 1). This meta-analysis received an exemption from the Institutional Review Boards of Seoul National University (IRB No. 2012/002-019). In addition, each study received IRB approval from its respective institution. Furthermore, some RCTs were registered at ClinialTrials.gov (accessed on 10 February 2022) or the International Clinical Trials Registry platform of the WHO before recruitment began. Principal investigators of individual RCTs willingly provided the requested raw data and related information for independent statistical reanalysis.

Despite similar aims and design, the six RCTs vary to a more or less considerable extent in the variables used in each study. Therefore, we evaluated data from the six studies and selected variables for meta-analysis based on the degree of overlap among studies. We only used the variables overlapped in two or more studies for meta-analysis and the corresponding interpretation of the results (Table 2).

### 2.3. Data Preprocessing and Transformation

Data preprocessing was conducted before the meta-analysis. First, we removed data that were not complete, such as “Quantity Not Sufficient (QNS).” Second, inequalities to mark specific cutoffs (ex. “> 500”) were substituted differently depending on the existence of observations beyond the cutoff for the particular variable. If there were no observations beyond the value in the inequality (ex. “>500” is the maximum observation) for a variable, that value was imputed in place of the inequality. When there were observations beyond the cutoff presented by the inequality, the imputed value was the mean of values within the inequality criteria before the following cutoff criterion. For instance, if there were two inequalities, “>500”, and “>600”, as well as observations 501, 503, 532, 640, and 700, “>500” would be imputed as 512 (mean of 501, 503, 532), while “>600” would be imputed as 670 (mean of 640 and 700). Finally, to analyze the intervention effects of PMS during the experimental period, we removed data without measurements at the start or end of the trial duration (“unpaired data”) and included only the paired data in the analysis.

Then, we additionally conducted a normalization process, which included a data transformation step, to reduce skewness and improve normality [17], and a standardization step. For the data transformation step, we considered two types of transformations to the raw observations: “square root” (taking square root on the expression levels) and “inverse normal” (taking the quantile of normal distribution using the rank of the expression levels). Among the two types, the optimal transformation type was set for each variable, based on the following process. First, skewness for each variable was calculated by combining skewness from individual studies, considering the sample size of individual studies as weight. If the absolute values of the two skewness values from the raw observations or “square-root” transformations are under 1, we selected the transformation whose skewness was the lesser of the two values. Otherwise, we selected “inverse normal” transformation for the optimal transformation. In detail, for “inverse normal” transformation, we first calculated (k−0.375)/(n+0.25) [18]. Then, we transformed it to quantiles of normal transformation, where *k* is the rank of the dataset and *n* is the sample size of the dataset from the relevant individual trial. The “inverse normal” transformation was used to reduce the skewness and effects of outliers, which could not be achieved through square-root transformation. After the transformation step was finished, we also performed a standardization step on the transformed expression levels to make effects of PMS from the individual studies comparable and, consequently, to combine the effects in the meta-analysis.

### 2.4. Data Analyses

The univariate analyses were first conducted to assess PMS effects based on different variables. To this end, the following linear effect model was used to handle repeated measures, as presented in Equation (1):

(1) $$y_{ijk}=\beta_{0k}+\beta_{1k}sex_{ik}+\beta_{2k}age_{ik}+\beta_{3k}wk_{ijk}+\beta_{4k}trt_{ik}+\beta_{5k}wk_{ijk}trt_{ik}+ind_{ik}+e_{ijk},$$

where *i* (=1, 2, …, *n_k_*) denotes subject id, *j* (=1,2) denotes the time point of measurements, and *k* (=1, 2, …, 6) denotes the index of individual studies. Thus, for each variable, $y_{ijk}$ is the level measured for the *i*th subject, at the *j*th time point, and in the *k*th study. Among the explanatory variables, *wk* is a binary week status, trt is a binary treatment status, and ind is an effect of the individual subject. Since we used a random intercept model to account for the potential heterogeneity among subjects, we set the ind variable as a random effect, and therefore, we assumed that $ind_{i}\sim N\left(0,\sigma_{1}{}^{2}\right)$ and $e_{ij}\sim N\left(0,\sigma_{2}{}^{2}\right)$. In the model, we regarded $\beta_{5k}$ to be the PMS effect on each variable because it implies the PMS effect on the treatment group at the end of the intervention.

The values of ${\hat{\beta }}_{5k}$ and their standard errors obtained from the univariate analyses were used as input information for the meta-analysis. The statistical results were pooled from all available studies for each variable to calculate the combined beta coefficient, standard error, and *p*-value. Then, we conducted Cochran’s Q test [19] to choose the meta-analysis model (fixed- or random-effect model) according to the presence of heterogeneity, as shown in Equation (2):

(2) $$Q=\overset{k}{\underset{i=1}{\sum }}W_{i}{\left(Y_{i}-\hat{\theta }\right)}^{2}\sim \chi^{2}\left(k-1\right)$$

where $W_{i}$ is the weight of the *i*th individual trial, represented as the reciprocal of the standard deviation obtained from the individual trials, $Y_{i}$ is the effect size observed for the *i*th individual trial, $\hat{\theta }$ is the pooled estimate, and *k* is the number of trials. If the test *p*-value is <0.05, the degree of heterogeneity is regarded as significant. In addition, the *I*^2^ statistic [20] was used to examine the quantitative measurement of statistical heterogeneity, as shown in Equation (3):

(3) $$I^{2}=100\%\times \frac{Q-df}{Q}$$

Finally, we conducted an overall and subgroup meta-analysis. Among the six studies included in this meta-analysis, four were performed in Asian subgroup meta-analysis, and the other two were in Western subgroup analysis, such as the USA and Russia. We categorized the studies as above because, as Asians accounted for a low percentage of the subjects studied in the USA (5.6%) and Russia (6.5%), it seemed reasonable to group the USA and Russia into the Western subgroup. In addition, because Korean studies account for two of the four Asian studies, a subgroup was created. Therefore, the subgroups are listed as follows: (1) Korean (2013 Korea and 2019 Korea), (2) Asian (2004 China, 2007 Japan, 2013 Korea, and 2019 Korea), and (3) Western (2015 USA and 2018 Russia).

All statistical analyses were performed using R 4.0.2, and a *p*-value ≤ 0.05 was considered statistically significant. The “lmer” function in the R package “lmerTest” was used to fit the linear mixed model. The heterogeneity test and meta-analysis were conducted using the “rma” function in the R package “metafor,” which can analyze random-effect (using restricted maximum likelihood estimator) and fixed-effect (using inverse-variance weighting method) analyses. When the interaction term between treatment and week of a variable was statistically significant in the overall group or the subgroup meta-analysis, it was regarded to have a significant effect induced by PMS and we interpreted its biological meaning.

## 3. Results

### 3.1. Characteristics of Trials and Subjects

The characteristics of trials and subjects of the six studies included in this meta-analysis are summarized in Table 1. They were conducted in east Asian countries, such as China (Study 1), Japan (Study 2), and Korea (Study 3 and 6), and in Western countries, such as USA (Study 4) and Russia (Study 5) between 2004 and 2019 in a randomized, double-blinded, placebo-controlled design for 6~8 week periods. The total number of subjects was increased to 886 after aggregating six intention-to-treat (ITT) populations. However, we included an 817 per-protocol (PP) population for the meta-analysis after removing 69 samples with incomplete data. All participants were healthy adults with habitually low fruit and vegetable intake, as identified by the recommended food score (RFS) [14,15,16,21]. The mean age was 38.7 ± 12.6 years and 37.3% (*n* = 305) of the sample was male.

### 3.2. Variable Selection

Table 2 lists the variables selected for meta-analysis according to the degree of overlap among studies. The variables were marked with an “O” or “X” based on the inclusion related to the study protocol. The last column represents the number of studies that included a corresponding variable in the protocol. For example, *β*-carotene was the most shared variable by being included in all studies, followed by homocysteine included in five studies; vitamin C, B_6_, B_12_, folate, and C-reactive protein (CRP) included in four studies; lutein, oxidized-low-density lipoprotein (ox-LDL), and Comet assay included in three studies; and *α*-tocopherol, *γ*-tocopherol, zeaxanthin, *α*-carotene, lycopene, malondialdehyde, Comet assay with H_2_O_2_ challenge, 8-hydroxy-2′-deoxyguanosine, and Short Form 36 questionnaire included in two studies. The selected variables were classified into three categories: oxidative stress, chemical scavenger, and quality of life.

### 3.3. Meta-Analysis Model Choice

Table 3 summarizes the results of Cochran’s Q test conducted to test the heterogeneity of the results among the different studies for choosing the most appropriate statistical model for the meta-analysis. The data were shown only for the six variables with statistically significant outcomes for the meta-analysis, and the results for variables without statistical significance are presented as supplementary information (Supplementary Table S1). In addition, we note that the data for vitamin B_12_ and *β*-carotene were normalized by inverse-normal transformation, while those for vitamin C, vitamin B_6_, folate, and ox-LDL were normalized by square-root transformation, based on the rule for the normalization process explained in the Materials and Methods section.

Substantial heterogeneity existed in the meta-analyses of vitamin B_6_ (Overall group, Asian subgroup, and Western subgroup), vitamin B_12_ (Overall group and Asian subgroup), folate (Overall group), and *β*-carotene (Overall group, Asian subgroup, and Western subgroup). Therefore, we employed a random-effect model for the meta-analysis of these variables. In contrast, a fixed-effect model was applied for the meta-analysis of the other variables, considering the homogeneous effects of PMS on them. Additional statistical heterogeneity evaluation was performed using the I^2^ statistic, which measures the relative magnitude of heterogeneity [20,22]. As expected, all variables employing the random-effect model based on Cochran’s Q test showed I^2^ > 50%, confirming substantial heterogeneity. Although one exception was observed in the overall meta-analysis of vitamin C with Cochran’s Q *p*-value = 0.095 and I^2^ = 52%, the other variables employing the fixed-effect model showed I^2^ < 50%. However, it was notable that the effects of vitamin C were homogeneous in the overall group and subgroups, based on the Cochran’s Q test.

### 3.4. Overall Effects of PMS on the Selected Variables

Table 4 displays results of the overall meta-analysis for the six variables with statistically significant outcomes. Results of insignificant variables are summarized in Supplementary Table S2. In Table 4, PMS exerted a significantly positive influence on increasing plasma vitamin C level in Study 5 ($\hat{\beta }$ = 0.5166, *p* = 0.008). Studies 2 and 4 also showed positive effects but did not reach statistical significance. Study 1 reported a contradictory effect, although its effect size was very small, without statistical significance ($\hat{\beta }$ = −0.0586, *p* = 0.6842). Therefore, the overall meta-analysis result remained marginally significant ($\hat{\beta }$ = 0.1692, *p* = 0.0797). Similar trends were found for the impact of PMS on changing plasma vitamin B_6_ and B_12_ levels. For vitamin B_6_, Studies 2, 4, and 5 all showed significantly positive results ($\hat{\beta }$ = 1.1703, *p* < 0.0001; $\hat{\beta }$ = 1.3509, *p* < 0.0001; and $\hat{\beta }$ = 0.7359, *p* = 0.001, respectively), while Study 1 showed a non-significant negative result. Consequently, the overall meta-analysis result was found to be positively significant ($\hat{\beta }$ = 0.8045, *p* = 0.0097). For vitamin B_12_, Studies 2 and 4 showed significantly positive results ($\hat{\beta }$ = 0.9322, *p* < 0.0001 and $\hat{\beta }$ = 0.564, *p* < 0.0001, respectively), while Study 5 showed a tendency of positive effect and Study 1 a non-significant negative result. As a result, the overall meta-analysis result was found to be positively significant ($\hat{\beta }$ = 0.4583, *p* = 0.0347). In the case of folate, the most consistent positive results were observed, with Studies 2 ($\hat{\beta }$ = 1.0468, *p* < 0.0001), 3 ($\hat{\beta }$ = 1.2046, *p* < 0.0001), 4 ($\hat{\beta }$ = 0.3212, *p* = 0.0869), and 5 ($\hat{\beta }$ = 0.6692, *p* = 0.007) being accounted for. Thus, the overall meta-analysis resulted in very large effect size ($\hat{\beta }$ = 0.8108, *p* = 0.0001). *β*-carotene is the most shared variable, but the results were inconclusive. Study 1 showed a non-significantly negative change, Studies 3 and 5 non-significantly positive changes, Study 6 a tendency of increase, and Studies 2 and 4 significant increase ($\hat{\beta }$ = 0.9339, *p* < 0.0001 and $\hat{\beta }$ = 0.8014, *p* < 0.0001, respectively). However, when the data were all combined for meta-analysis, the resulting beta coefficient and *p*-value indicated a globally significant positive effect of PMS on plasma *β*-carotene level ($\hat{\beta }$ = 0.3631, *p* = 0.0307). Finally, PMS influence on plasma ox-LDL level was non-significantly negative in Studies 3 and 6, while non-significantly positive in Study 5. However, surprisingly, the *p*-value was improved when data were combined for the overall meta-analysis.

**Figure 1:**
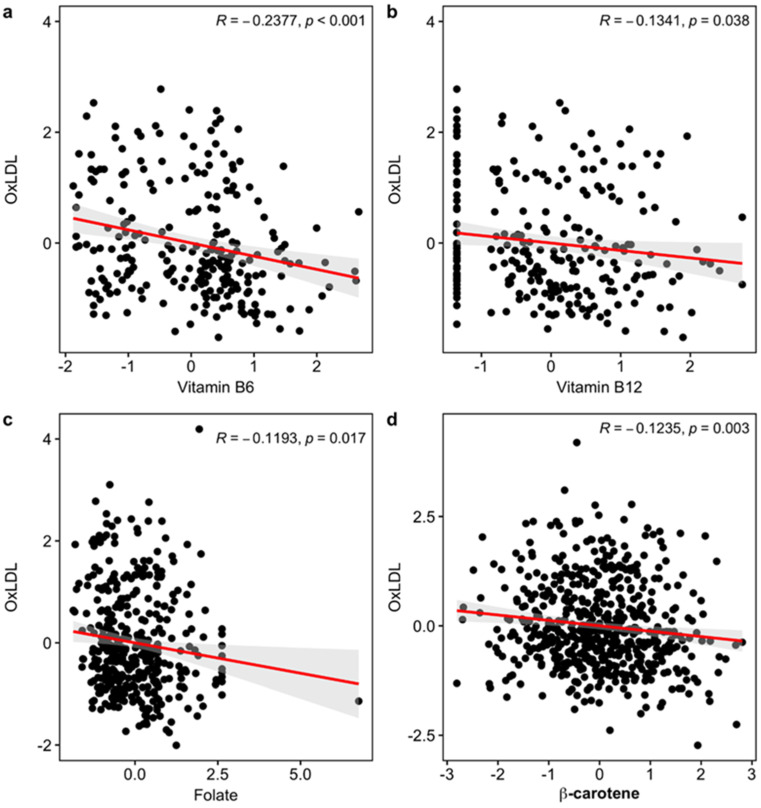
Scatterplot between ox-LDL and each of the four variables that are significantly associated with ox-LDL: (**a**) Vitamin B_6_, (**b**) Vitamin B_12_, (**c**) Folate, and (**d**) *β*carotene. Regression lines between the variables were drawn as red lines, and 95% confidence intervals of the mean were drawn as gray regions. We only put Pearson’s correlation coefficients and the corresponding *p*-values here.

Next, among the six significant markers in the meta-analysis, we found significantly negative associations of vitamin B_6_ ($r_{p}$ = −0.2377, *p* < 0.001; $r_{s}$ = −0.2433, *p* < 0.001), B_12_ ($r_{p}$ = −0.1341, *p* = 0.038; $r_{s}$ = −0.1386, *p* = 0.032), folate ($r_{p}$ = −0.1193, *p* = 0.017; $r_{s}$ = −0.1149, *p* = 0.022), and *β*-carotene ($r_{p}$ = −0.1235, *p* = 0.003; $r_{s}$ = −0.1243, *p* = 0.003) with plasma ox-LDL (Figure 1). Here, $r_{p}$ is coefficient of the Pearson’s correlation, and $r_{s}$ is coefficient of the Spearman’s rank correlation to reduce the effects of outliers. Those values and the corresponding *p*-values are similar to each other, which means that there is not any severe effect of outliers.

### 3.5. Subgroup Analysis

**Figure 2:**
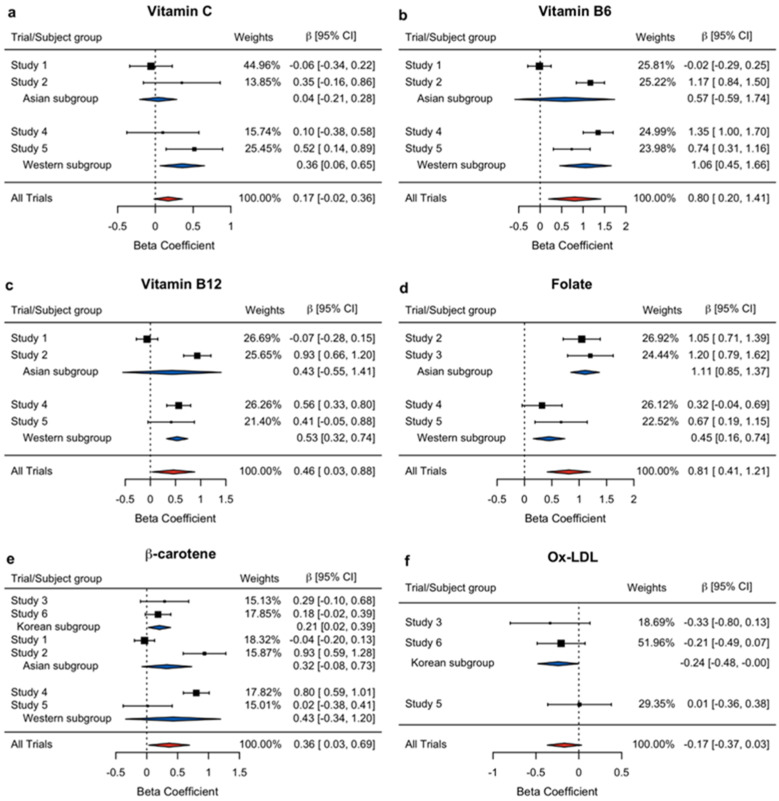
Forest plots of the beta coefficients and their associated 95% confidence intervals for the effects of PMS on exogenous oxidant scavengers and oxidative stress: (**a**) Vitamin C, (**b**) Vitamin B_6_, (**c**) Vitamin B_12_, (**d**) Folate, (**e**) *β*-carotene, and (**f**) Ox-LDL. The individual effects are demonstrated as black squares, and pooled effects as red (for the overall) and blue (for the subgroup) diamonds. The box sizes reflect the relative weighting of each study for contribution to the meta-analysis. CI: confidence interval, Ox-LDL: oxidized low-density lipoprotein.

The overall meta-analysis results support the positive influence of the PMS to increase vitamin C, B_6_, B_12_, folate, and *β*-carotene, and decrease ox-LDL, but with non-significant *p*-values for vitamin C and ox-LDL. Therefore, considering that regional similarity may impact the sensitivity to the PMS intervention, we first performed the subgroup meta-analyses by region. The results are presented in Table 4 and in Figure 2 as forest plots, demonstrating the pooled effect as a diamond with a horizontal line for the beta estimate and their 95% confidence interval, respectively. Thus, the size is proportional to the weight percentage used in the meta-analysis. Overall, all selected variables proved to have significant results in at least one subgroup meta-analysis. While vitamin C ($\hat{\beta }$ = 0.3570, *p* = 0.0177), vitamin B_6_ ($\hat{\beta }$ = 1.0566, *p* = 0.0006), vitamin B_12_ ($\hat{\beta }$ = 0.5327, *p* < 0.0001), and folate ($\hat{\beta }$ = 0.4488, *p* = 0.0024) were found to be positive and significant in the Western subgroup, the Asian subgroup showed a significant positive influence only on the plasma folate level ($\hat{\beta }$ = 1.1095, *p* < 0.0001). In the meantime, the Korean subgroup showed significant effects of PMS on *β*-carotene ($\hat{\beta }$ = 0.2057, *p* = 0.0263) and Ox-LDL ($\hat{\beta }$ = −0.2401, *p* = 0.0489).

## 4. Discussion

This meta-analysis of six RCTs comprising 817 PP participants from different regional populations helped forge a better understanding of the effectiveness of PMS in improving oxidative defense compared to the individual RCTs. The main findings are the following: (1) Our meta-analysis could conclude that PMS remarkably influenced plasma vitamin C, B_6_, B_12_, folate, *β*-carotene, and ox-LDL, while the univariate analysis in individual studies found conflicting results. (2) The antioxidant potential of PMS may be derived from the increase in the plasma levels of free radical scavenging vitamins, including vitamin B_6_, B_12_, folate, and *β*-carotene. (3) There were some regional differences in the sensitivity to PMS supplementation. Likewise, our results supported the general knowledge of how meta-analysis could overcome the lack of power, settle controversies arising from studies with conflicting findings, and derive conclusions not directly addressed by individual research [23,24].

When we ran a scan on “meta-analysis for multivitamins” on the PubMed search engine, we found that there were 36 published papers. The most frequent issue was about the benefits of multivitamin use during pregnancy and prenatal development (18 cases), followed by those related to risk reduction in chronic diseases, such as cancers (4 cases), circulation diseases (4 cases), eye diseases (4 cases), cognitive diseases (2 cases), infections (2 cases), hip fracture (1 case), and mortality (1 case). Unfortunately, these previous meta-analyses estimated a clinical endpoint or surrogate marker that is more difficult to observe than clinical markers, limiting the success of meta-analysis [25]. Unlike the previous studies, the current study performed a meta-analysis based on the plasma exposure level of the free radical scavenging nutrients and the relevant clinical biomarkers to examine the PMS ability to react to oxidative stress. Although meta-analysis could not replace a well-designed, adequately powered RCT, it can overcome the limitation of smaller trials and generate a single best estimate by pooling groups based on a priori defined criteria for inclusion [26]. For example, in the case of ox-LDL, none of the individual studies showed a significant influence of PMS, but we could generate a remarkable estimate in a subgroup meta-analysis.

Overall, our meta-analysis results showed that PMS might have a beneficial effect in enhancing free radical scavenging nutrient levels in plasma, as shown in the cases of vitamin C, B_6_, B_12_, folate, and *β*-carotene, and in suppressing oxidative stress, as indicated by reduced ox-LDL in plasma. Subsequent association analysis suggested that increased plasma levels of antioxidant nutrients were involved in suppressing oxidative stress, based on a confirmed correlation between plasma vitamins and ox-LDL levels. However, vitamin C was an exception in this study. This result is consistent with a previous study that reviewed the relationship between dietary vitamin C intake and plasma levels of vitamin C [27]. It was suggested that various confounding factors might influence plasma levels of vitamin C, including body size, smoking, bioavailability, absorption condition, and stress. For example, 100 mg/day of ascorbic acid is adequate to saturate the slope of the relation between intake and blood [28].

The strength of our study is worth mentioning. Most importantly, unlike meta-analysis based on aggregating study data, our meta-analysis has the advantage of using individual subject data from independently performed RCTs, which are often considered the gold standard for meta-analysis [29]. Individual participant data may provide a clearer picture of the subgroup. Therefore, we could reliably investigate subgroup effects of interventions [30]. Secondly, we avoid publication bias via using the full dataset from all available studies, regardless of their publication status. Ferro et al. [31] compared meta-analysis estimates based on the published reports with those based on individual participant pooled analyses and concluded that published data yielded less precise summary estimates, probably due to publication bias. Thirdly, since a simple pooling of study results might ignore the precision and thus yield misleading summary estimates, we computed weighted averages by employing a random-effect model or a fixed-effect model [32]. Finally, we quantified heterogeneity in data analysis by Cochran Q test, confirming the result by I^2^ statistic. Here, statistical heterogeneity refers to variation in the true effect sizes between the studies, rather than the variation due to sampling error [33]. Substantial heterogeneity was found in the overall meta-analysis of plasma vitamin B_6_, B_12_, folate, and *β*-carotene, and thus we applied random-effect models to calculate pooled estimates of the outcomes. Although this approach usually makes little difference to the final estimate, the overall meta-analysis resulted in large effect sizes for vitamin B_6_, B_12_, folate, and *β*-carotene. In the meantime, vitamin C and ox-LDL had a relatively low heterogeneity in the overall meta-analysis. We thus applied fixed-effect models, in which the pooled effect estimates the common true effect size.

On the other hand, our study also has some limitations. Multivitamin supplements from natural sources tend to be preferred over many other nutritional supplements distributed in the market, probably due to the presence of cofactors known as phytonutrients [34]. However, we could not add any phytochemicals as an exposure biomarker in the current study due to limited resources. Thus, it is highly recommended for future research to include new variables, such as phytochemicals and their metabolites in plasma to investigate the effect of PMS more precisely.

## 5. Conclusions

In conclusion, our meta-analysis supports a general benefit of PMS in alleviating oxidative stress by providing free radical scavenging nutrients in the general population with a habitually low intake of fruit and vegetables. In future studies, we will adopt metabolomics technologies to include the analysis of phytochemicals and their metabolites in plasma and expand the concept of the benefits of plant-based vitamin supplementation.

## Figures and Tables

**Table 1:** Characteristics of individual RCTs ^†^ included in the current meta-analysis.

Studies	Data Source	Country	Year	Period (wk)	RFS ^‡^	PP ^§^ Subjects (ITT ^¶^ Subjects)	Age (SD ^#^)	Sex (M/F)
Study 1	Unpublished	China	2004	8	<12 (23)	297 (334)	33.2 (11.0)	123/174
Study 2	Unpublished	Japan	2007	8	<12 (23)	116 (126)	43.0 (14.2)	40/76
Study 3	Kim et al. [16]	Korea	2013	8	<37 (47)	80 (90)	43.2 (9.2)	38/42
Study 4	Unpublished	USA	2015	6	<12 (23)	120 (120)	33.7 (12.1)	57/63
Study 5	Isakov et al. [14]	Russia	2018	8	<12 (23)	120 (120)	49.2 (7.5)	21/99
Study 6	Kang et al. [15]	Korea	2019	8	<37 (47)	84 (96)	39.9 (11.3)	26/58
Total						817 (886)	38.7 (12.6)	305/512

^†^ RCT: randomized placebo-controlled trial, ^‡^ RFS: recommended food score, ^§^ PP: Per-protocol, ^¶^ ITT: Intention-to-treat, ^#^ SD: standard deviation.

**Table 2:** Selected variables for performing a meta-analysis. The mark “O” denotes the presence of the variable in the protocol of the corresponding study, and “X” if not. The total count represents the number of “O” s.

Categories	Variables	Individual Studies						Total Count
		# 1	# 2	# 3	# 4	# 5	# 6	
Chemical Scavenger	*α*-tocopherol, plasma	O	X	X	O	X	X	2
	*γ*-tocopherol, plasma	O	X	X	O	X	X	2
	Vitamin C, plasma	O	O	X	O	O	X	4
	Vitamin B_6_, plasma	O	O	X	O	O	X	4
	Vitamin B_12_, plasma	O	O	X	O	O	X	4
	Folate, plasma	X	O	O	O	O	X	4
	Lutein, plasma	O	O	X	X	X	O	3
	Zeaxanthin, plasma	X	O	X	X	X	O	2
	*α*-carotene, plasma	O	X	X	X	X	O	2
	*β*-carotene, plasma	O	O	O	O	O	O	6
	Lycopene, plasma	O	X	X	X	X	O	2
Oxidative Damage	Homocysteine, plasma	O	O	O	O	O	X	5
	CRP ^†^, plasma	O	X	O	O	X	O	4
	MDA ^‡^, plasma	X	X	O	X	X	O	2
	Ox-LDL ^§^, plasma	X	X	O	X	O	O	3
	Comet assay, PBMC	O	X	O	X	X	O	3
	Comet assay, PBMC H_2_O_2_ challenge	X	X	O	X	X	O	2
	8-OHdG ^#^, urine	O	X	X	X	X	O	2
QOL	Short Form 36 questionnaire	O	X	O	X	X	X	2

^†^ CRP: C-reactive protein, ^‡^ MDA: malondialdehyde, ^§^ Ox-LDL: oxidized low-density lipoprotein, **^¶^** PBMC: peripheral blood mononuclear cells, ^#^ 8-OHdG: 8-hydroxy-2′-deoxyguanosine.

**Table 3:** Meta-analysis models of overall and subgroup analysis for individual variables with significant results.

Categories	Variable (Optimal Transformation)	Meta-Analysis	Model	Heterogeneity	
				Cochran’s Q (*p*-Value)	I^2^ (%)
Chemical scavenger	Vitamin C (Square-root)	Overall	Fixed-effect	6.37 (0.0950)	52.00
		Asian sub	Fixed-effect	1.90 (0.1682)	47.35
		Western sub	Fixed-effect	1.82 (0.1772)	45.07
	Vitamin B_6_ (Square-root)	Overall	Random-effect	47.97 (<0.0001)	92.32
		Asian sub	Random-effect	29.81(<0.0001)	96.65
		Western sub	Random-effect	4.76 (0.0292)	78.98
	Vitamin B_12_ (Inverse-normal)	Overall	Random-effect	35.13 (<0.0001)	89.58
		Asian sub	Random-effect	32.37 (<0.0001)	96.91
		Western sub	Fixed-effect	0.32 (0.5730)	<0.01
	Folate (Square-root)	Overall	Random-effect	12.58 (0.0056)	75.21
		Asian sub	Fixed-effect	0.33 (0.5648)	<0.01
		Western sub	Fixed-effect	1.29 (0.2564)	22.36
	*β*-carotene (Inverse-normal)	Overall	Random-effect	54.70 (<0.0001)	90.11
		Korean sub	Fixed-effect	0.22 (0.6357)	<0.01
		Asian sub	Random-effect	25.38 (<0.0001)	90.34
		Western sub	Random-effect	11.82 (0.0006)	91.54
Oxidative Damage	Ox-LDL (Square-root)	Overall	Fixed-effect	1.43 (0.4882)	<0.01
		Korean sub	Fixed-effect	0.21 (0.6436)	<0.01

**Table 4:** Overall and subgroup meta-analysis results for significant variables.

Category	Variable	Relevant Trials		Meta-Analysis		
			Weights (%)	Beta Coefficient	Standard Error	*p*-Value
Chemical Scavenger	Vitamin C	Study 1	44.96	−0.0586	0.1440	0.6842
		Study 2	13.85	0.3504	0.2595	0.1784
		Study 4	15.74	0.0988	0.2434	0.6855
		Study 5	25.45	0.5166	0.1914	0.0080
		Overall	100	0.1692	0.0966	0.0797
		Asian sub	-	0.0377	0.1259	0.7647
		Western sub	-	0.3570	0.1505	0.0177
	Vitamin B_6_	Study 1	25.81	−0.0182	0.1391	0.8959
		Study 2	25.22	1.1703	0.1674	<0.0001
		Study 4	24.99	1.3509	0.1778	<0.0001
		Study 5	23.98	0.7359	0.2188	0.0010
		Overall	100	0.8045	0.3110	0.0097
		Asian sub	-	0.5724	0.5942	0.3354
		Western sub	-	1.0566	0.3072	0.0006
	Vitamin B_12_	Study 1	26.69	−0.0662	0.1088	0.5432
		Study 2	25.65	0.9322	0.1377	<0.0001
		Study 4	26.26	0.5640	0.1213	<0.0001
		Study 5	21.40	0.4148	0.2353	0.0805
		Overall	100	0.4583	0.2170	0.0347
		Asian sub	-	0.4294	0.4992	0.3896
		Western sub	-	0.5327	0.1078	<0.0001
	Folate	Study 2	26.92	1.0468	0.1728	<0.0001
		Study 3	24.44	1.2046	0.2128	<0.0001
		Study 4	26.12	0.3212	0.1857	0.0869
		Study 5	22.52	0.6692	0.2440	0.0070
		Overall	100	0.8108	0.2024	0.0001
		Asian sub	-	1.1095	0.1341	<0.0001
		Western sub	-	0.4488	0.1478	0.0024
	*β*-carotene	Study 1	15.13	−0.0354	0.0834	0.6718
		Study 2	17.85	0.9339	0.1755	<0.0001
		Study 3	18.32	0.2889	0.1985	0.1495
		Study 4	15.87	0.8014	0.1063	<0.0001
		Study 5	17.82	0.0156	0.2023	0.9388
		Study 6	15.01	0.1826	0.1047	0.0851
		Overall	100	0.3631	0.1681	0.0307
		Korean sub	-	0.2057	0.0926	0.0263
		Asian sub	-	0.3248	0.2063	0.1154
		Western sub	-	0.4274	0.3924	0.2762
Oxidative Damage	Ox-LDL (Square root)	Study 3	18.69	−0.3342	0.2371	0.1627
		Study 5	51.96	0.0085	0.1892	0.9644
		Study 6	29.35	−0.2063	0.1422	0.1507
		Overall	100	−0.1672	0.1025	0.1029
		Korean sub	-	−0.2401	0.1219	0.0489

## Data Availability

Data are available by request to the authors.

## Supplementary Materials

The following supporting information can be downloaded at: https://www.mdpi.com/article/10.3390/nu14061170/s1, Supplementary Table S1: Meta-analysis models of overall and subgroup analysis for individual variables without significant results.; Supplementary Table S2: Overall and subgroup meta-analysis results for variables without significant results.
