# Supplementary material for: Meta-Analysis of Randomized Clinical Trials Evaluating Effectiveness of a Multivitamin Supplementation against Oxidative Stress in Healthy Subjects
Source: Nutrients. 2022 Mar 10;14(6):1170. doi: 10.3390/nu14061170 (PMC8955918; doi:10.3390/nu14061170)
Supplement: Supplementary file 1 [file nutrients-14-01170-s001.zip › nutrients-1613921-supplementary.pdf]

Supplementary Table S1. Meta-analysis models of overall and subgroup analysis for individual variables without significant results.

Categories	Variable (Optimal transformation)	Meta-analysis	Model	Heterogeneity	
				Cochran's Q (*p*-value)	I^2 (%)
**Chemical scavenger**	α-tocopherol (Square-root)	Overall	Random-effect	9.2623 (0.0023)	89.20
	γ-tocopherol (Square-root)	Overall	Random-effect	9.5850 (0.0020)	89.57
	Lutein (Square-root)	Overall	Random-effect	9.1555 (0.0103)	78.41
	Zeaxanthine (Square-root)	Overall	Fixed-effect	0.4525 (0.5012)	< 0.01
	α-carotene (Square-root)	Overall	Fixed-effect	0.7399 (0.3897)	< 0.01
	Lycopene (Square-root)	Overall	Fixed-effect	2.0456 (0.1526)	51.11
Oxidative Damage	Homocysteine (Inverse normal)	Overall	Fixed-effect	4.5180 (0.3404)	26.29
		Asian sub	Fixed-effect	3.5578 (0.1688)	46.29
		Western sub	Fixed-effect	0.7165 (0.3973)	< 0.01
	C-Reactive Protein (Inverse normal)	Overall	Fixed-effect	0.2525 (0.9687)	< 0.01
		Korean sub	Fixed-effect	0.0110 (0.9166)	< 0.01
		Asian sub	Fixed-effect	0.1848 (0.9117)	< 0.01
	MDA (Square-root)	Overall	Fixed-effect	0.0218 (0.8827)	< 0.01
	Alkaline Tail Intensity (Square-root)	Overall	Fixed-effect	1.2002 (0.5488)	< 0.01
		Korean sub	Fixed-effect	0.1654 (0.6842)	< 0.01
	Alkaline Tail Length (Square-root)	Overall	Fixed-effect	3.9017 (0.1422)	48.67
		Korean sub	Fixed-effect	2.2252 (0.1358)	55.06
	Alkaline Tail Moment (Square-root)	Overall	Fixed-effect	2.2721 (0.3211)	22.32
		Korean sub	Fixed-effect	0.5560 (0.4559)	< 0.01
	H_2O_2 Tail Intensity (Inverse normal)	Overall	Fixed-effect	1.6834 (0.1945)	40.60
	H_2O_2 Tail Length (Inverse normal)	Overall	Fixed-effect	0.0026 (0.9596)	< 0.01
	H_2O_2 Tail Moment (Inverse normal)	Overall	Fixed-effect	1.4207 (0.2333)	29.61
	Urine 8-OHdG (Square-root)	Overall	Fixed-effect	0.5941 (0.4408)	< 0.01
QOL	SF-36 Questionnaire (Inverse normal)	Overall	Fixed-effect	0.0509 (0.8215)	< 0.01

Supplementary Table S2. Overall and subgroup meta-analysis results for variables without significant results.

Category	Variable	Relevant trials	Meta-analysis			
			Weights (%)	Beta coefficient	standard error	*p*-value
**Chemical scavenger**	**α-tocopherol**	Study 1	53.15	-0.0138	0.0869	0.8738
		Study 4	46.85	0.5659	0.1695	0.0012
		Overall	100	0.2578	0.2893	0.3729
	γ-tocopherol	Study 1	53.32	0.1476	0.0924	0.1113
		Study 4	46.68	-0.5227	0.1958	0.0088
		Overall	100	-0.1653	0.3344	0.6210
	Lutein	Study 1	38.51	-0.0087	0.0835	0.9171
		Study 2	24.72	-0.5104	0.1905	0.0091
		Study 6	36.77	-0.3155	0.0972	0.0017
		Overall	100	-0.2455	0.1423	0.0844
	Zeaxanthine	Study 2	19.34	-0.0160	0.1932	0.9341
		Study 6	80.66	-0.1607	0.0946	0.0932
		Overall	100	-0.1327	0.0850	0.1183
	α-carotene	Study 1	56.32	-0.0360	0.0723	0.6185
		Study 6	43.68	-0.1301	0.0821	0.1169
		Overall	100	-0.0771	0.0543	0.1553
	Lycopene	Study 1	71.77	-0.0302	0.0996	0.7617
		Study 6	28.23	-0.2983	0.1588	0.0639
		Overall	100	-0.1059	0.0844	0.2095
Oxidative Damage	Homocysteine	Study 1	40.01	0.0209	0.0968	0.8292
		Study 2	29.41	-0.2574	0.1129	0.0245
		Study 3	10.13	-0.1457	0.1924	0.4512
		Study 4	13.48	-0.0957	0.1668	0.5674
		Study 5	6.97	-0.3375	0.2319	0.1482
		Overall	100	-0.1185	0.0612	0.0529
		Asian sub	-	-0.1032	0.0686	0.1327
		Western sub	-	-0.1781	0.1354	0.1883
	C-Reactive Protein	Study 1	56.95	0.0235	0.0997	0.8138
		Study 3	5.55	-0.0289	0.3195	0.9281
		Study 4	22.65	-0.0446	0.1581	0.7782
		Study 6	14.86	-0.0681	0.1952	0.7281
		Overall	100	-0.0084	0.0752	0.9107
		Korean sub	-	-0.0574	0.1666	0.7302
		Asian sub	-	0.0022	0.0855	0.9799
	MDA	Study 3	36.15	-0.0597	0.2351	0.8003
		Study 6	63.85	-0.0163	0.1769	0.9268
		Overall	100	-0.0320	0.1414	0.8210
	Alkaline Tail Intensity	Study 1	63.82	0.0797	0.1533	0.6036
		Study 3	15.18	-0.0822	0.3143	0.7940
		Study 6	20.99	-0.2500	0.2673	0.3523
		Overall	100	-0.0141	0.1225	0.9084
		Korean sub	-	-0.1796	0.2036	0.3778
	Alkaline Tail Length	Study 1	62.58	0.0697	0.1516	0.6462
		Study 3	16.31	0.0816	0.2970	0.7841
		Study 6	21.11	-0.5082	0.2610	0.0550
		Overall	100	-0.0504	0.1199	0.6745

		Korean sub	-	-0.2512	0.1961	0.2001
	Alkaline Tail Moment	Study 1	59.97	0.0897	0.1520	0.5554
		Study 3	15.90	-0.0541	0.2952	0.8552
		Study 6	24.13	-0.3376	0.2396	0.1627
		Overall	100	-0.0363	0.1177	0.7579
		Korean sub	-	-0.2250	0.1860	0.2265
	H_2O_2 Tail Intensity	Study 3	48.31	-0.3477	0.2955	0.2411
		Study 6	51.69	0.1856	0.2857	0.5177
		Overall	100	-0.0721	0.2054	0.7257
	H_2O_2 Tail Length	Study 3	47.04	-0.1480	0.3045	0.6283
		Study 6	52.96	-0.1692	0.2870	0.5573
		Overall	100	-0.1592	0.2089	0.4458
	H_2O_2 Tail Moment	Study 3	44.91	-0.3833	0.3124	0.2218
		Study 6	55.08	0.1184	0.2821	0.6756
		Overall	100	-0.1069	0.2094	0.6095
	Urine 8-OHdG	Study 1	79.75	0.0038	0.1167	0.9743
		Study 6	20.25	-0.1961	0.2316	0.3998
		Overall	100	-0.0367	0.1042	0.7249
QOL	SF-36 Questionnaire	Study 1	84.38	0.0182	0.1070	0.8654
		Study 3	15.62	0.0793	0.2487	0.7508
		Overall	100	0.0277	0.0983	0.7777
